# Source-Term Release Behavior and Mechanisms of Non-Metallic Leaching Parameters from Coal Gangue: COD, Sulfate, and Fluoride

**DOI:** 10.3390/toxics14070635

**Published:** 2026-07-21

**Authors:** Siqi Xu, Yiyong Xu, Yufei Yang, Qifei Huang, Qingqi Die

**Affiliations:** 1 College of Water Science, Beijing Normal University, Beijing 100085, China; 202231470036@mail.bnu.edu.cn; 2 State Key Laboratory of Environmental Criteria and Risk Assessment, Chinese Research Academy of Environmental Sciences, Beijing 100012, China; xuyiyong25@mails.ucas.ac.cn (Y.X.); yangyf@craes.org.cn (Y.Y.); 3 State Environmental Protection Key Laboratory of Hazardous Waste Identification and Risk Control, Chinese Research Academy of Environmental Sciences, Beijing 100012, China

**Keywords:** coal gangue, organic matter, sulfate, fluoride, leaching release mechanisms

## Abstract

Long-term open-air storage of coal gangue (CG) can generate leachates containing oxidizable components contributing to chemical oxygen demand (COD), together with sulfate (SO_4_^2−^) and fluoride (F^−^), yet their source-term behavior remains poorly understood. In this study, batch and column leaching tests were conducted on multi-source CG samples and combined with ToF-SIMS (surface compositional mapping), solid-phase TOC gradient leaching, and PHREEQC-XGBoost-SHAP modeling (coupled geochemical–machine learning analysis). Batch-leachate COD levels ranged from 4.6 to 68.0 mg/L, while SO_4_^2−^ and F^−^ concentrations reached maxima of approximately 317 and 1.29 mg/L, respectively. During column leaching, COD levels and SO_4_^2−^ concentrations were highest at low liquid-to-solid ratios (L/S) and subsequently decreased, with maximum initial values of 186.6 and 3074 mg/L, respectively, whereas F^−^ exhibited delayed and persistent release at approximately 0.3–2.3 mg/L. Solid-phase TOC did not directly predict the COD response, and ToF-SIMS revealed aliphatic organic fragments associated with aluminosilicate surfaces that weakened or were redistributed after leaching. The COD–DOC discrepancy further indicated that dissolved organic matter alone could not fully explain the COD response, although the possible contribution of inorganic reducing species requires direct verification. Within the modeled framework, the sulfate source-term coefficient accounted for 69.4–76.9% of the modeled influence at L/S = 0.5–2.0 L/kg, while the influence of the HFO surface complexation increased during later leaching. In contrast, the F^−^ source-term coefficient remained dominant over L/S = 0.5–10.0 L/kg, accounting for 97.0–97.9% of the modeled influence. These findings support parameter- and stage-specific monitoring and pollution control at CG disposal sites.

## 1. Introduction

Global coal production, consumption, trade, and coal-fired power generation remained at high levels in 2024, with total coal production reaching approximately 8.77 billion tons [1]. Coal gangue (CG) is one of the major solid wastes generated during coal mining and washing, accounting for approximately 20% of total coal production. It is characterized by large generation volumes and long storage periods [2,3]. Consequently, large quantities of CG are commonly stockpiled in open-air dumps in mining areas. Under rainfall infiltration, surface runoff, and groundwater contact, continuous water–rock interactions can promote the migration of soluble salts, organic or reducing components, and constituents derived from sulfur- and fluorine-bearing minerals into surrounding water systems [4,5]. Because CG disposal sites are usually open systems that persist for long periods, their leachates may enter nearby surface water or groundwater and alter the chemical composition of receiving waters [6,7].

Previous studies on the environmental impacts of CG have mainly focused on the occurrence, leaching, migration, and ecological risks of heavy metals or potentially toxic elements [8,9,10]. However, CG also contains residual coal-derived organic or reducing substances, sulfur-bearing minerals, and fluorine-bearing minerals, which may generate leachates characterized by elevated levels of chemical oxygen demand (COD), sulfate (SO_4_^2−^), and fluoride (F^−^) [11]. These non-metallic leaching parameters often occur at relatively high concentrations and exhibit strong mobility [12], and may directly affect water quality around mining areas. Nevertheless, their dynamic release behaviors and interfacial control mechanisms in CG leaching systems remain insufficiently understood.

Residual coal matter in CG contains aromatic compounds, including naphthalene-, phenanthrene-, and fluorene-related compounds, which may increase the concentration of organic components in leachates. Previous studies reported that COD concentrations in CG leachates can reach 8.41–12.76 mg/L [13]. However, owing to high dilution ratios and short contact times, these static tests merely represent a highly conservative, short-term screening scenario. In reality, field monitoring of actual water bodies severely impacted by coal mining and the long-term weathering of CG indicates that actual COD levels can reach as high as 183.0 mg/L [14]. This significant discrepancy demonstrates that standard static tests severely underestimate the long-term, dynamic leaching potential of organic substances under natural weathering and cumulative rainfall infiltration. Elevated BOD and COD levels have also been observed in groundwater near coal mining areas [15]. Sulfate is mainly derived from soluble sulfate minerals, sulfur-bearing minerals such as pyrite, and their weathering products, and its release is closely associated with the formation of acid mine drainage [16,17,18]. In CG, fluorine predominantly exists in inorganic forms, with minor amounts occurring as water-soluble or organically bound fractions. The primary fluorine-bearing phases include independent minerals (e.g., fluorapatite, fluorite, and fluorphlogopite) and various clay minerals (e.g., kaolinite and illite) [19,20]. The occurrence modes of fluorine are mainly characterized by the isomorphic substitution of hydroxyl groups (OH^−^) within the crystal lattices of aluminosilicates, as well as specific physical and chemical adsorption on mineral surfaces [21]. Under varying leaching conditions, these exchangeable and surface-adsorbed fluorine fractions can be gradually released into the surrounding systems through water–rock interactions. Elevated F^−^ concentrations have also been reported in groundwater in some coal mining areas [22]. Therefore, in addition to heavy metals, COD-related oxidizable components and the release of SO_4_^2−^, and F^−^ should also be incorporated into environmental risk assessments of CG disposal sites.

Standard batch leaching procedures, such as HJ 299 and TCLP, are widely used to evaluate the environmental leaching behavior of CG [23]. These static tests, conducted under fixed pH or liquid-to-solid ratio conditions, are useful for evaluating the potential release levels of pollutants. However, rainfall-driven leaching in actual disposal sites is a long-term mass-transfer process that evolves with the cumulative liquid-to-solid ratio (L/S). Static leaching tests are therefore limited in describing pollutant release at different leaching stages [24]. For organic pollution, many studies have inferred the composition of dissolved organic matter from the spectral characteristics of leachates [25]. Less attention has been paid to the initial occurrence and aqueous migration of organic components from the perspective of the CG solid matrix and mineral interfaces. Under aqueous conditions, the surfaces of hydrophilic minerals, such as silicates, carbonates, and metal oxides, generally exhibit strong hydration and adsorption properties, thereby influencing the adsorption, enrichment, and migration of low-molecular-weight organic compounds [26]. For SO_4_^2−^ and F^−^, previous studies have used sequential extraction, isotope tracing, or geochemical modeling to identify their sources and release conditions [11,19,23]. In actual leaching systems, however, mineral dissolution, precipitation, and surface adsorption/desorption often occur simultaneously. Distinguishing the relative roles of these processes at different leaching stages remains difficult when relying only on simple correlation or saturation-index analysis.

In recent years, the Shapley additive explanations (SHAP) method has been widely used to interpret the relative contributions of input variables to machine-learning model predictions [24,25]. Direct PHREEQC sensitivity analysis can evaluate the effects of selected geochemical parameters, but it is less efficient for summarizing stage-dependent parameter importance in multi-parameter systems with nonlinear interactions [27,28]. In this study, PHREEQC was retained as the geochemical constraint model, whereas XGBoost was used as a surrogate model trained on PHREEQC simulation outputs. SHAP analysis was then applied to the surrogate model to identify the relative influence of different reaction parameters on simulated release concentrations at different L/S stages. This coupled PHREEQC-XGBoost-SHAP approach therefore provides an interpretable supplement to conventional geochemical modeling rather than a replacement for PHREEQC.

Based on these considerations, this study focuses on the source-term release behavior and controlling mechanisms of representative non-metallic leaching parameters from CG, including COD-related oxidizable components, SO_4_^2−^ and F^−^. These parameters are associated with residual coal-derived organic or reducing substances, sulfur-bearing minerals, and fluorine-bearing minerals, respectively, and can reflect important release characteristics beyond those of heavy metals during water–rock interactions. First, batch leaching tests were conducted on CG samples from different sources to screen their release potentials. Representative samples with different release intensities were then selected for column leaching tests to investigate their L/S-dependent dynamic release behavior. To clarify the controlling mechanisms, time-of-flight secondary ion mass spectrometry (ToF-SIMS) surface analysis, solid-phase TOC gradient leaching tests, and PHREEQC-XGBoost-SHAP analysis were combined to examine the interfacial occurrence, aqueous leachability, and geochemical controls of these COD-related components and ions. This study aims to reveal the source-term release mechanisms of representative non-metallic leaching parameters from CG and to support site-specific water-quality risk assessment and stage-specific pollution control at CG disposal sites.

## 2. Materials and Methods

### 2.1. Sample Collection and Pretreatment

**Figure 1:**
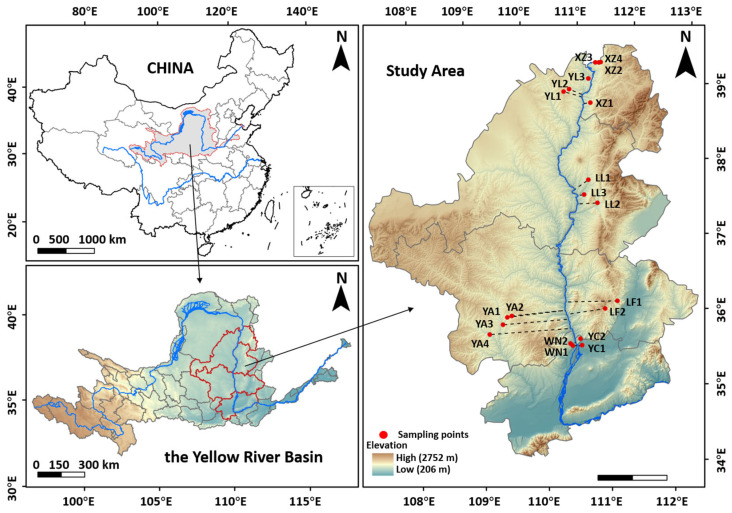
Sampling locations map. Note: Blue lines indicate rivers; the red line indicates the boundary of the Yellow River Basin; gray dashed lines indicate the straight-line distances from the sampling points to the Yellow River.

CG is a solid by-product generated during coal mining and washing and is generally composed of residual coal and mineral impurities separated from raw coal. In this study, 20 CG samples from different sources were collected from representative coal mining areas in the middle reaches of the Yellow River Basin, and the sampling locations are shown in Figure 1. The coal seams in this region were mainly formed during the Carboniferous–Permian period and have similar geological origins and diagenetic backgrounds, which provides a basis for the comparability and regional representativeness of the collected samples. In addition, the mining processes in the study area are generally similar, reducing the influence of large process-related differences on sample properties.

Sampling sites were located in newly deposited areas of CG disposal sites at each coal mining enterprise to reduce the influence of long-term natural weathering on the initial properties of the samples. At each disposal site, 15 sampling grids were established, and approximately 2 kg of CG was collected from a depth of 0–30 cm at the center of each grid. Subsamples collected from the same disposal site were thoroughly mixed to prepare a composite sample of approximately 30 kg. The samples were sealed, transported to the laboratory, and used for subsequent leaching experiments, physicochemical analyses, and solid-phase characterization. Before the experiments, the composite samples were crushed, sieved, and homogenized, and representative subsamples were obtained according to the requirements of different experiments. All leaching experiments were conducted in triplicate to ensure the reproducibility of the results.

### 2.2. Leaching Protocols

#### 2.2.1. Batch Leaching Test

To evaluate the potential release levels of COD-related oxidizable components, SO_4_^2−^, and F^−^ from CG samples of different sources, batch leaching tests were conducted on the 20 CG samples according to HJ/T 299-2007 [29]. This method is commonly used to identify the leaching toxicity of solid wastes and can serve as a screening test for the release potential of target constituents under acidic conditions. The extractant was deionized water adjusted to pH 3.20 ± 0.05 using a mixed acid solution of sulfuric acid and nitric acid at a mass ratio of 2:1, as specified in HJ/T 299-2007 [29]. The COD level and F^−^ concentration of the extractant blank were below their respective method detection limits, and the SO_4_^2−^ blank concentration was deducted from the reported leaching data. The L/S was set at 10 L/kg. The samples were tumbled at 30 r/min for 18 h. After leaching, the suspensions were filtered through 0.45 μm membranes, and the filtrates were used to determine COD levels and the concentrations of SO_4_^2−^ and F^−^.

#### 2.2.2. Dynamic Column Leaching Test

To investigate the dynamic release behavior of the target parameters as a function of the L/S during long-term leaching, column leaching tests were conducted according to US EPA Method 1314. Based on the HJ/T 299-2007 [29] batch leaching results, one sample was selected from each of the spatially separated YA, LL, and YL source areas (Figure 1). Sample selection considered both source differences and the release characteristics of the target parameters. The selected samples exhibited measurable but contrasting release concentrations, allowing comparison of their dynamic release behavior and controlling mechanisms under different release intensities.

Three replicate columns were prepared for each sample and operated in an up-flow mode. The CG samples were crushed, sieved, and homogenized to a particle size of <2 mm to minimize the influence of particle-size variation on the leaching results. Before continuous leaching, each packed column was pre-saturated with deionized water from the bottom upward at a low flow rate to avoid preferential flow and to remove trapped air from the pore spaces. The pre-saturation step was continued until the column was fully wetted and a stable outflow was observed. After pre-saturation, the column leaching experiment was started under the same up-flow mode. Leaching continued until the cumulative L/S reached 10.0 L/kg. Leachates were collected at predefined L/S stages, filtered through 0.45 μm membranes, and analyzed for COD levels and the concentrations of SO_4_^2−^ and F^−^.

#### 2.2.3. Solid-Phase Total Organic Carbon Gradient Leaching Test

To analyze the relationship between the COD response and solid-phase total organic carbon (TOC) content in CG, seven CG samples from the same source but with different solid-phase TOC contents were selected for batch leaching tests. The samples were crushed, sieved, and homogenized, and deionized water was used as the extractant. Based on the release results at low L/S stages in Method 1314, the L/S ratio was set at 1.5 L/kg. The samples were tumbled at 30 r/min for 18 h. After leaching, the suspensions were filtered through 0.45 μm membranes, and the filtrates were used to determine COD levels and dissolved organic carbon (DOC) concentrations.

### 2.3. Analytical Methods

#### 2.3.1. Liquid-Phase Analysis

After collection, each leachate sample was thoroughly mixed, and only a representative aliquot required for chemical analysis was filtered through a 0.45 μm membrane filter before analysis. This filtration step was used to remove suspended particles and coal dust from the analyzed aliquots. The filtered aliquots were used to determine COD levels and the concentrations of SO_4_^2−^, F^−^, and DOC.

COD was determined using the dichromate method according to HJ 828-2017 (Water quality—Determination of the chemical oxygen demand—Dichromate method) [30]. F^−^ was determined using the fluoride reagent spectrophotometric method according to HJ 488-2009 (Water quality—Determination of fluoride—Fluoride reagent spectrophotometric method) [31]. SO_4_^2−^ was determined using the barium chromate spectrophotometric method according to HJ/T 342-2007 (Water quality—Determination of sulfate—Barium chromate spectrophotometric method) [32]. Parallel samples were included during analysis, and calibration curves were established using standard solutions, with correlation coefficients greater than 0.999. DOC in the filtered leachates was measured using a total organic carbon analyzer and was used as a quantitative indicator of the dissolved organic matter fraction.

#### 2.3.2. Solid-Phase Mineral Composition and Surface Analysis

X-ray diffraction (XRD) was used to analyze the mineral composition of the CG samples, and the identified major mineral phases were used as an important basis for selecting candidate minerals in the PHREEQC model. To investigate the occurrence characteristics of surface components on CG particles and their changes before and after leaching, time-of-flight secondary ion mass spectrometry (ToF-SIMS, PHI nanoTOF II, Physical Electronics, Inc., Chanhassen, MN, USA) was used to analyze the surfaces of samples before and after Method 1314 leaching. Before ToF-SIMS analysis, the CG samples were ground with an agate mortar and stored in clean glass sample vials. During sample preparation, the powder was spread onto conductive adhesive tape and introduced into the instrument pre-vacuum chamber. The samples were evacuated for approximately 40–60 min until the vacuum level met the requirement for transfer into the analysis chamber. The samples were then introduced into the analysis chamber for ToF-SIMS measurement. During analysis, selected particle surface areas were scanned in both positive and negative ion modes to obtain inorganic elemental signals, including Al, Si, K, Ca, S, and F, as well as organic fragment signals, such as C_2_H^−^, C_3_H_5_^−^, C_3_H_7_^−^, and C_4_H_7_^−^. By comparing the spatial distributions and relative intensities of different ion signals before and after leaching, the surface distribution characteristics of organic and inorganic components and their leaching responses were analyzed.

### 2.4. PHREEQC-XGBoost-SHAP Coupled Model for Quantitative Identification of Release Mechanisms

#### 2.4.1. PHREEQC Geochemical Model

Based on the Method 1314 column leaching results, a source-term model coupled with PHREEQC was developed to simulate changes in SO_4_^2−^ and F^−^ concentrations with increasing cumulative L/S. The concentrations measured at L/S = 0.2 L/kg were used as the initial anchor values, representing the initial leaching concentrations under the column leaching conditions. A first-order exponential function was used to describe the gradual approach of the source-term concentrations from their initial values toward the prescribed long-term concentrations during leaching. Here, the prescribed long-term concentrations refer to the concentration values set in the source-term equation to represent the later-stage concentration level approached as L/S increases. They were used only as model parameters and should not be interpreted as directly measured field equilibrium concentrations.

Three parameters, $k_{SO_{4}^{2-}}$, $k_{F^{-}}$, and *p_m_*_,*HFO*_, were selected as the main model inputs. The parameters $k_{SO_{4}^{2-}}$ and $k_{F^{-}}$ are effective first-order coefficients defined on the cumulative L/S scale. $k_{SO_{4}^{2-}}$ characterizes the attenuation of readily releasable sulfate-related components during leaching, whereas $k_{F^{-}}$ characterizes the approach of the F^−^ source-term concentration toward its long-term value. Thus, these parameters describe the effective evolution of source-term concentrations with cumulative L/S. The parameter *p_m_*_,*HFO*_ represents the logarithm of the total amount of reactive hydrous ferric oxide (HFO) surface sites and characterizes the surface complexation capacity available for SO_4_^2−^ and F^−^ adsorption–desorption reactions. The source-term equation, parameter units and ranges, and HFO site definitions are provided in Text S1.

PHREEQC was subsequently used to simulate carbonate buffering, aqueous speciation, and surface complexation under the experimental conditions. The minteq.v4.dat database was used, with the temperature set to 25 °C and the initial pH set to 6.0. Calcite was defined as the principal carbonate-buffering phase using the EQUILIBRIUM_PHASES module. Weak and strong HFO surface sites were defined using the SURFACE module to represent the adsorption–desorption of SO_4_^2−^ and F^−^ on reactive metal oxide surfaces. After each calculation, total aqueous F and S(VI) were extracted as model outputs, and S(VI) was converted to the corresponding SO_4_^2−^ concentration. Detailed PHREEQC settings are provided in Text S2.

#### 2.4.2. XGBoost Surrogate Model and SHAP Contribution Analysis

Latin hypercube sampling (LHS) was used to generate 10,000 parameter combinations. These parameter sets were then used for batch PHREEQC simulations to obtain F^−^ and SO_4_^2−^ concentrations at different L/S stages. The PHREEQC simulation results were used as the training dataset for a multi-output XGBoost surrogate model, with the sampled parameters as inputs and the simulated concentrations as outputs.

The purpose of constructing the XGBoost surrogate model was to emulate the PHREEQC outputs and to support efficient interpretation of stage-dependent parameter effects. The surrogate model did not replace the PHREEQC geochemical model. Instead, it provided a computationally efficient approximation of PHREEQC-simulated concentrations for subsequent SHAP analysis. The surrogate model showed high predictive performance. For SO_4_^2−^, the R^2^ values were 0.991 and 0.995 for the validation and test sets, respectively. The corresponding values for F^−^ were 0.975 and 0.977. These results indicate that the surrogate model accurately reproduced the PHREEQC-simulated concentrations and was suitable for subsequent SHAP analysis. The complete R^2^, RMSE, and MAE results are provided in Table S1.

The trained XGBoost surrogate model was interpreted using SHAP to calculate the relative influence of different parameters on the predicted SO_4_^2−^ and F^−^ concentrations at each L/S stage. To avoid interference from the initial concentration anchor point, SHAP analysis was performed only for L/S = 0.5–10.0 L/kg. Spearman rank correlation analysis was further used to examine the relationships between key parameters and simulated concentrations. It should be noted that SHAP values represent the relative influence of input parameters on PHREEQC-constrained surrogate predictions. They should not be interpreted as direct reaction rates or absolute mass-flux contributions in the actual reaction processes. The XGBoost hyperparameters and SHAP/Spearman calculation procedures are provided in Texts S3 and S4.

## 3. Results and Discussion

### 3.1. Screening Leaching Risks of COD, SO_4_^2−^ and F^−^ from Multi-Source CG

**Figure 2:**
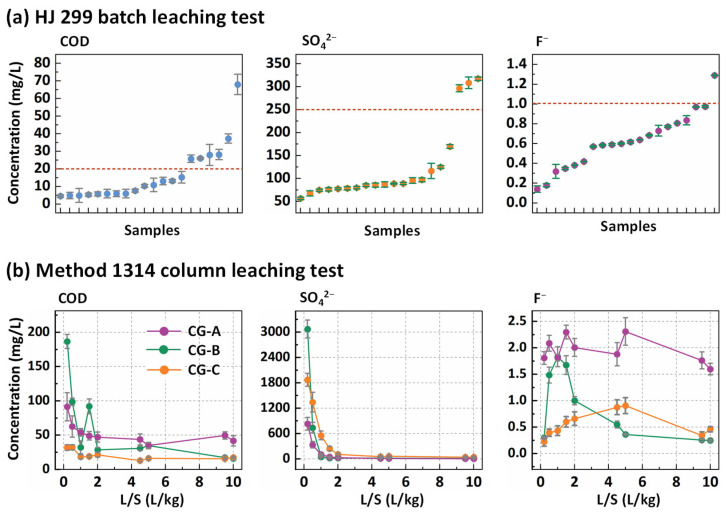
Leaching risks and L/S-dependent release behavior of COD, SO_4_^2−^ and F^−^ from CG: (**a**) batch leaching results for 20 CG samples; and (**b**) L/S-dependent release profiles obtained from the Method 1314 column leaching tests. The dashed lines in panel (**a**) indicate the water-quality reference limits adopted from GB 3838-2002. Error bars in panel (**b**) represent the standard deviations of three replicate experiments (*n* = 3).

Before discussing the leaching results, the mineralogical background of the CG samples was examined. XRD analysis showed that the principal mineral phases were quartz (SiO_2_), Pyrite (FeS_2_), muscovite (KAl_2_Si_3_AlO_10_(OH)_2_), kaolinite (Al_2_Si_2_O_5_(OH)_4_), Anorthite ((Ca,Na)(Al,Si)_2_Si_2_O_8_), Calcite ((Ca,Mg)CO_3_), and Sodium Slilcate (Na_4_SiO_4_) (Figure S1), which is consistent with the typical mineral composition of CG reported in previous studies [33]. These aluminosilicate minerals may provide important mineral surfaces for the occurrence and interaction of organic fragments, sulfate-related species, and fluoride during water–rock interaction. Batch leaching tests were conducted on 20 CG samples according to HJ 299-2007 [29] to evaluate the potential release of representative non-metallic leaching parameters from CG. The concentrations of COD, SO_4_^2−^, and F^−^ in the leachates were measured, and the results are shown in Figure 2a.

The leached COD concentrations ranged from 4.6 to 68 mg/L. Most samples were below the COD limit of 20 mg/L for Class III surface water in the Environmental Quality Standards for Surface Water (GB 3838-2002) [34], which was used here as a risk reference value. However, some samples exceeded this value under the acidic leaching conditions of HJ 299. The highest COD concentration was 68 mg/L, about 3.4 times the reference value. This suggests that some CG samples may release high levels of oxidizable components under acidic or unfavorable environmental conditions. Because COD represents the overall response of oxidizable substances, an increase in COD cannot be simply interpreted as an increase in dissolved organic carbon. Therefore, DOC measurements and solid-phase interfacial characterization are needed to further identify its sources.

The leached SO_4_^2−^ concentrations were mainly in the range of 55–170 mg/L. A few samples exceeded the reference value of 250 mg/L under acidic leaching conditions, with the highest concentration reaching approximately 317 mg/L. Sulfur in CG may come from soluble sulfate minerals, sulfur-bearing minerals such as pyrite, and their weathering products. Under acidic leaching conditions, these sulfur-bearing components may enter the aqueous phase through dissolution or oxidation, leading to elevated SO_4_^2−^ concentrations in the leachate.

The leached F^−^ concentrations ranged from 0.14 to 1.29 mg/L. Most samples were below the F^−^ limit of 1.0 mg/L for Class III surface water, which was used as a reference value. Only the sample with the highest concentration exceeded this value under acidic leaching conditions. Compared with COD and SO_4_^2−^, F^−^ showed lower overall concentrations in the batch leaching tests. However, some samples approached or exceeded the reference value, indicating that fluorine-bearing components in CG still have release potential.

Overall, clear differences were observed among the 20 CG samples in terms of COD levels and the leaching concentrations of SO_4_^2−^ and F^−^. These differences may be related to variations in sample source, mineralogical composition, residual coal matter content, and the occurrence forms of sulfur- and fluorine-bearing components. COD levels varied greatly among samples, SO_4_^2−^ generally showed relatively high leaching concentrations, and F^−^ was mostly below the reference value but still posed a potential release risk in some samples. These results suggest that the source-term assessment of CG should not focus only on heavy metals or potentially toxic elements. Non-metallic leaching parameters related to residual coal matter, sulfur-bearing minerals, and fluorine-bearing minerals should also be considered when identifying constituents of concern for subsequent water-quality risk assessment. Based on the batch leaching results, three samples with measurable but contrasting release concentrations were selected from the YA, LL, and YL source areas for the Method 1314 column leaching tests. This selection enabled further comparison of their L/S-dependent release behavior and controlling mechanisms across different release intensities and sample origins.

### 3.2. L/S-Dependent Dynamic Release Behavior

To examine pollutant release during long-term leaching, three representative samples, CG-A, CG-B, and CG-C, were selected for Method 1314 column leaching tests. The L/S-dependent release curves of COD, SO_4_^2−^, and F^−^ are shown in Figure 2b.

COD levels were high at low L/S and then decreased in all three samples. CG-B showed the highest initial COD level, reaching 186.6 mg/L at L/S = 0.2 L/kg, which was higher than the maximum value observed in the HJ/T 299-2007 [29] batch leaching test. This difference can be mainly attributed to the much lower L/S condition at the initial stage of the column leaching test, where released COD-related oxidizable components were concentrated in a smaller leachate volume. With increasing L/S, the COD concentration of CG-B decreased markedly. CG-A had an initial COD concentration of 91.4 mg/L, which later remained within 41–54 mg/L. CG-C showed lower COD concentrations, ranging from 15.6 to 32.2 mg/L. These results suggest that COD-related oxidizable components were mainly released during the early leaching stage, although the release intensity varied among samples.

SO_4_^2−^ followed a more typical initial flushing pattern. CG-A, CG-B, and CG-C reached maximum SO_4_^2−^ concentrations of 830.5, 3074, and 1872 mg/L, respectively, at L/S = 0.2 L/kg. The concentrations then decreased rapidly with increasing L/S and remained relatively low in the later stages. This pattern suggests that readily releasable sulfate components in CG can quickly enter the aqueous phase during the initial contact with flowing water.

In contrast, F^−^ exhibited delayed and persistent release. The F^−^ concentration in CG-A ranged from approximately 1.6 to 2.3 mg/L throughout the column experiment. In CG-B, the F^−^ concentration initially increased to 1.8 mg/L at L/S < 1 L/kg and subsequently decreased to approximately 0.3 mg/L, whereas that in CG-C remained within the range of 0.40–0.83 mg/L. The pH values of the column leachates (Table S2) ranged from 8.03 to 8.62 for CG-A, from 7.19 to 7.36 for CG-B, and from 7.77 to 8.03 for CG-C. Under neutral-to-mildly alkaline conditions, competition between OH^−^ and surface-associated F^−^ may promote the desorption or exchange of some releasable fluoride species, partly explaining the higher and more persistent F^−^ concentrations in CG-A. However, the relatively low F^−^ concentrations in CG-C indicate that pH alone cannot explain the differences among the samples. The available XRD analysis did not detect discrete F-bearing mineral phases, such as fluorite, fluorapatite, or fluorophlogopite, indicating that any such phases, if present, were below the XRD detection limit. ToF-SIMS showed partial spatial co-localization of the F^−^ and Ca-related signals, suggesting that some fluorine may have occurred in association with Ca-bearing components. However, this spatial association cannot conclusively identify a specific Ca-bearing mineral phase, such as fluorite or fluorapatite. Fluorine may also occur in clay and other aluminosilicate minerals through structural incorporation or surface adsorption [19,20,21]. Therefore, the differences in F^−^ release among the samples likely resulted from the combined effects of pH-dependent interfacial reactions, fluorine occurrence forms, and the initial inventory of releasable fluorine.

Overall, COD levels and SO_4_^2−^ concentrations were relatively high at low L/S and decreased as leaching proceeded, whereas F^−^ persisted over a wider L/S range. This difference suggests that CG may generate short-term high inputs of COD-related oxidizable components and SO_4_^2−^ during the early stage of rainfall infiltration, while F^−^ requires more attention during long-term leaching. The three samples also differed in release intensity and curve shape. CG-B showed a high initial COD response and SO_4_^2−^ release, CG-A showed higher and more persistent F^−^ release, and CG-C showed a low overall COD response but strong initial SO_4_^2−^ release. These results indicate that the COD response and the release of SO_4_^2−^ and F^−^ were not synchronous. Their behavior may be jointly affected by occurrence forms, interfacial accessibility, and aqueous geochemical reactions. Therefore, the following sections discuss the mechanisms from two aspects: the interfacial occurrence and aqueous mobility of COD-related organic/reducing components, and the dissolution-precipitation and adsorption–desorption processes controlling SO_4_^2−^ and F^−^.

### 3.3. Interfacial Controls on the COD Response

The column leaching results showed that COD levels were high at low L/S and then gradually decreased. This pattern suggests that the components responsible for the COD response were not uniformly distributed throughout the CG particles. Instead, they were more likely associated with particle surfaces or connected pores that were readily accessible to flowing water. To further identify the sources of the COD response and the related interfacial controls, ToF-SIMS surface analysis and solid-phase total organic carbon (TOC) gradient leaching tests were used to examine the relationships among solid-phase TOC, leachate DOC, and COD levels.

**Figure 3:**
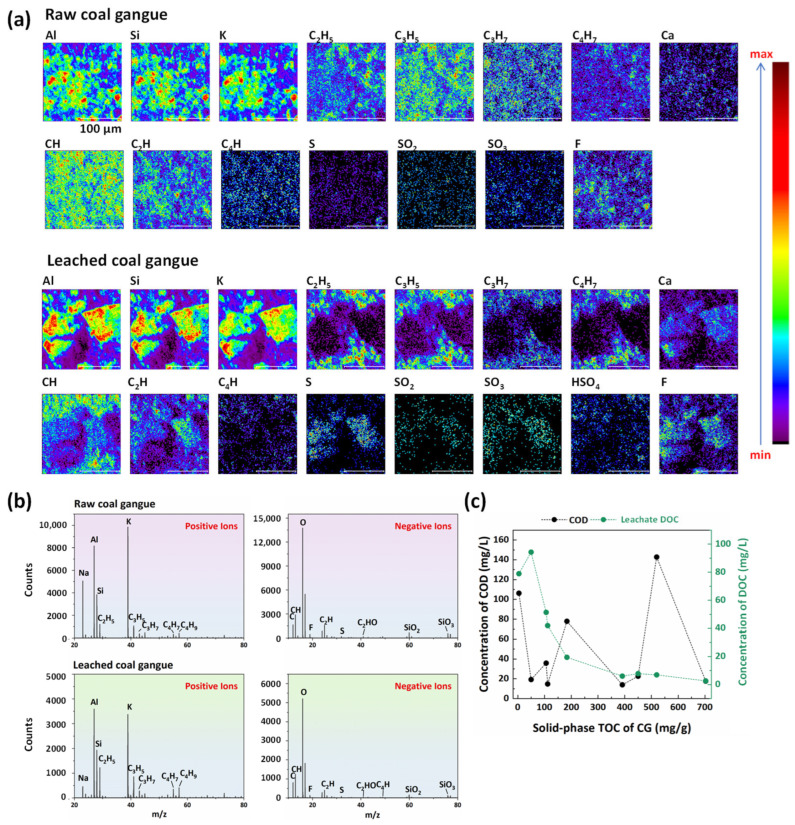
ToF-SIMS characterization and TOC-gradient leaching results of CG. (**a**) ToF-SIMS ion maps of raw and leached CG particles, the white dashed line indicates the area analyzed by surface mapping. (**b**) ToF-SIMS positive- and negative-ion mass spectra of raw and leached CG particles. (**c**) Relationships among solid-phase TOC, leachate DOC, and COD levels.

The ToF-SIMS results indicated organic–inorganic associations on CG particle surfaces. As shown in Figure 3a,b, strong mineral framework signals, including Al, Si, K, and Na, were detected in the unleached samples. Carbon-containing fragments, such as C_2_H_5_^−^, C_3_H_5_^−^, and C_3_H_7_^−^, were also observed, indicating the coexistence of low-molecular-weight organic components and aluminosilicate mineral surfaces. Surface mapping further showed spatial overlap between some aliphatic organic fragments and Al, Si, and K signals. This suggests that hydrophilic low-molecular-weight organic fragments were adsorbed or weakly bound to aluminosilicate surfaces. These surfaces commonly contain hydroxyl groups, which can generate variable surface charge depending on solution conditions. Together with other charged sites, these hydroxyl-bearing surface groups provide adsorption sites and enhance the affinity of mineral surfaces for hydrophilic organic fragments [22,35]. When leachate entered interparticle pores and contacted these mineral surfaces, weakly bound hydrophilic organic fragments could enter the aqueous phase through desorption, dissolution, or surface detachment. This process may have contributed to the high COD response at low L/S. After leaching, Al, Si, and K signals still showed clear spatial distributions, whereas some organic fragment signals weakened or became redistributed. In particular, the distribution range and relative intensity of aliphatic fragments such as C_3_H_5_^−^ and C_3_H_7_^−^ decreased. These changes suggest that some weakly bound hydrophilic organic components on mineral surfaces had aqueous mobility during water–rock interaction and could enter the aqueous phase during the early leaching stage.

It should be noted that ToF-SIMS provides surface-sensitive and semi-quantitative information. Therefore, the weakened organic fragment signals after leaching cannot be directly interpreted as quantitative removal of organic matter, as they may also be affected by surface redistribution, matrix effects, and changes in mineral surface exposure. Together with the increase in leachate COD, these observations support the possible aqueous mobility of some surface-associated oxidizable components, but do not provide conclusive evidence for their complete removal from particle surfaces.

The solid-phase TOC gradient leaching results further showed that the total organic carbon content of CG did not directly determine leachate DOC concentration. As shown in Figure 3c, some samples with low solid-phase TOC had relatively high DOC concentrations. In contrast, when solid-phase TOC increased to approximately 391–703 mg/g, leachate DOC remained low, ranging from 2.6 to 6.1 mg/L. This result indicates that the total amount of organic carbon in CG is not equivalent to the release potential of soluble organic carbon. In samples with low solid-phase TOC, a small amount of organic matter may have been mainly associated with hydrophilic mineral surfaces, making it more accessible to water and easier to release. In samples with high solid-phase TOC, more organic carbon may have occurred as residual coal matter or hydrophobic organic matrices. These forms have lower aqueous accessibility and are less likely to enter the aqueous phase in large amounts during short-term leaching.

The relationships among COD, solid-phase TOC, and leachate DOC were more complex. In some low-TOC samples, both COD and DOC levels were high. For example, in the sample with a solid-phase TOC content of 3.8 mg/g, COD and DOC reached 106.3 and 78.9 mg/L, respectively. This indicates that soluble or desorbable organic matter can directly contribute to COD. However, some high-TOC samples showed low DOC levels but markedly elevated COD levels. For example, in the sample with a solid-phase TOC content of 519 mg/g, COD and DOC were 142.8 and 6.9 mg/L, respectively. This suggests that COD was not controlled only by DOC. Because COD reflects the overall consumption of oxidants by oxidizable substances, it is not specific to dissolved organic matter. Previous studies and standard COD methodologies have shown that inorganic reducing species, such as Fe^2+^, sulfide, sulfite, and other reduced sulfur species, can also consume oxidants and affect COD measurements [36]. Therefore, these species provide a possible explanation for part of the discrepancy between COD and DOC observed in this study. Targeted measurements of Fe speciation and reduced sulfur species would be needed to distinguish their relative contributions.

Overall, the COD response in CG leachates was jointly affected by interfacially accessible organic components and possible inorganic reducing substances. During the early leaching stage, flowing water preferentially contacted weakly bound organic fragments and some reducing components on hydrophilic mineral surfaces and in connected pores, resulting in high COD levels. As these readily releasable components were depleted, COD levels decreased. In contrast, organic carbon hosted in hydrophobic residual coal matter may be abundant, but it does not necessarily have high short-term aqueous mobility. Therefore, solid-phase TOC content alone may not reliably indicate the release potential of COD-related oxidizable components. Leachate DOC, surface organic–inorganic associations, and possible inorganic reducing components should be considered together when interpreting COD responses in CG leachates.

### 3.4. Geochemical Controls on SO_4_^2−^ and F^−^ Release

The column leaching results showed that SO_4_^2−^ was rapidly eluted at low L/S, whereas F^−^ exhibited delayed and persistent release. ToF-SIMS analysis further revealed differences in their surface occurrence and leaching responses. Before leaching, sulfur-containing signals, including S, SO_2_^−^, and SO_3_^−^, were locally enriched and partially overlapped with the Al and Si signals. After leaching, HSO_4_^−^ was detected on the particle surfaces, suggesting changes in surface sulfur species during leaching. This signal may reflect the oxidation of sulfur-bearing components by dissolved oxygen during the leaching experiment or the transformation and redistribution of surface-adsorbed sulfate species under dynamic flow conditions. In contrast, the F^−^ signal did not decrease markedly after leaching and was partially co-localized with Ca. These observations suggest that the two ions differed in their interfacial occurrence and release characteristics.

During leaching, SO_4_^2−^ and F^−^ are commonly controlled by mineral dissolution-precipitation and surface adsorption–desorption processes [37,38]. Based on the PHREEQC-XGBoost-SHAP model described in Section 2.4, the simulated concentration ranges of SO_4_^2−^ and F^−^ covered the observations from the Method 1314 column leaching experiment. This result indicates that the model captured the main geochemical processes under the experimental conditions. SHAP analysis was then used to quantify the relative contributions of different reaction parameters to SO_4_^2−^ and F^−^ release at different L/S stages. This allowed the stage-dependent effects of mineral release and surface desorption to be identified. The dynamic changes in SHAP feature importance across L/S stages are provided in Figure S2.

**Figure 4:**
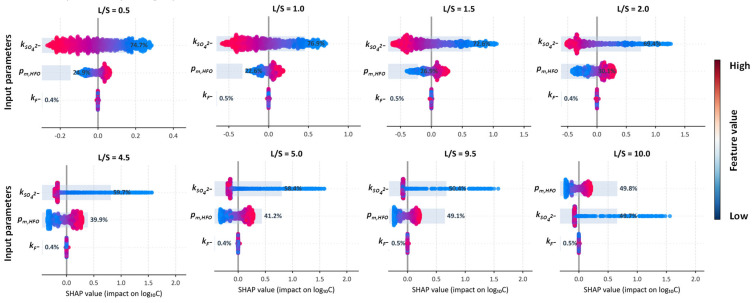
PHREEQC-XGBoost-SHAP analysis of stage-dependent controls on SO_4_^2−^ release. Note: Light-blue shaded bars indicate normalized global feature importance, with percentages showing the relative contribution of each parameter at the corresponding L/S ratio.

**Figure 5:**
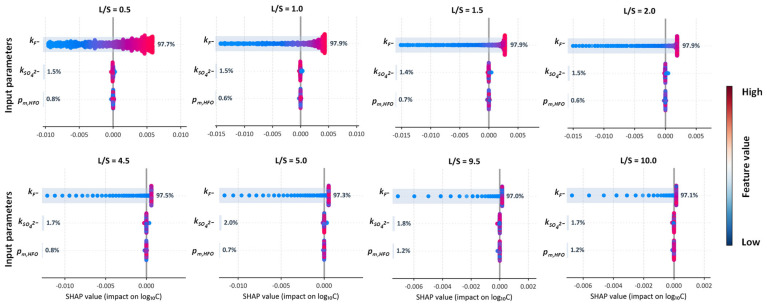
PHREEQC-XGBoost-SHAP analysis of stage-dependent controls on F^−^ release. Note: Light-blue shaded bars indicate normalized global feature importance, with percentages showing the relative contribution of each parameter at the corresponding L/S ratio.

For SO_4_^2−^, SHAP analysis showed a stage-dependent change in parameter influence. During the rapid flushing stage (L/S = 0.5–2.0 L/kg), $k_{SO_{4}^{2-}}$, which characterizes the attenuation of readily releasable sulfate-related components, dominated the predictions, with a relative influence of 69.4–76.9% (Figure 4). This result is consistent with the rapid decrease in SO_4_^2−^ concentration observed during column leaching. As L/S increased, the relative influence of $k_{SO_{4}^{2-}}$ decreased to 49.7% at L/S = 10.0 L/kg, whereas that of *p_m_*_,*HFO*_ increased to 49.8%. Within the modeled framework, this shift indicates a decreasing influence of the initial sulfate source term and an increasing influence of HFO surface complexation during the later leaching stages. For F^−^, $k_{F^{-}}$ remained the dominant parameter over L/S = 0.5–10.0 L/kg, with a relative influence of 97.0–97.9% (Figure 5). This parameter characterizes the approach of the F^−^ source-term concentration toward its prescribed long-term value as cumulative L/S increases. Its consistently high influence agrees with the persistent F^−^ release observed in the column experiment.

Spearman correlation analysis further supported the SHAP interpretation. With increasing L/S, the correlation between SO_4_^2−^ concentration and $k_{SO_{4}^{2-}}$ weakened from −0.96 at L/S = 0.5 L/kg to −0.28 at L/S = 10.0 L/kg. In contrast, the correlation between SO_4_^2−^ concentration and *p_m_*_,*HFO*_ strengthened, reaching 0.84 at L/S = 10.0 L/kg. F^−^ concentration remained strongly correlated with $k_{F^{-}}$, with correlation coefficients of up to 1.00. Together, these results indicate that the modeled SO_4_^2−^ concentrations became progressively more sensitive to HFO surface complexation at higher L/S, whereas the modeled F^−^ concentrations remained primarily sensitive to the source-term coefficient throughout the analyzed stages.

### 3.5. Environmental Implications

The batch leaching and column leaching tests provided different types of information for evaluating the source-term release risk of CG. The HJ/T 299-2007 [29] batch test was conducted at L/S = 10 L/kg under acidic conditions and was used as a conservative screening method for release potential. In contrast, the Method 1314 column test was performed under up-flow percolation conditions and generated concentration profiles as cumulative L/S increased. Therefore, the higher initial COD concentration observed in the column test, especially at L/S = 0.2 L/kg, can be mainly attributed to the much lower L/S at the early leaching stage, where released components were concentrated in a smaller leachate volume. The batch test is useful for comparing the release potential among multi-source CG samples, whereas the column test better reflects dynamic leachate generation during rainfall infiltration or water percolation through CG piles. Thus, the two methods are complementary for screening, monitoring design, and stage-specific risk management at CG disposal sites.

Pollutant release from CG changes during rainfall-driven leaching under long-term storage conditions. This study shows that COD, SO_4_^2−^, and F^−^ differ markedly in both release stage and release persistence. Therefore, pollution control at CG disposal sites should consider the dominant pollutants at different leaching stages. During the early stage of storage or backfill utilization, COD and readily releasable SO_4_^2−^ may rapidly enter the aqueous phase at low L/S. Thus, COD and SO_4_^2−^ in early-stage leachates should be closely monitored for newly deposited CG or during the initial stage of backfill utilization. As leaching proceeds, the initial flushing effect gradually weakens; however, F^−^ may still show persistent release, and SO_4_^2−^ may also maintain a certain level of late-stage release due to surface desorption. Therefore, for long-term CG disposal sites, the continuous release of F^−^ and SO_4_^2−^ should still be considered and incorporated into long-term monitoring and stage-specific pollution control.

This study also indicates that COD in CG leachates should be interpreted with caution. In routine monitoring, COD is commonly used as an indicator of oxidizable organic pollution. However, COD in CG leachates reflects the overall oxidant consumption by oxidizable substances and may not originate solely from dissolved organic matter. Therefore, using COD alone to evaluate the release intensity of organic pollutants from CG may lead to bias. In addition, high solid-phase TOC content does not necessarily correspond to high DOC or COD levels in leachates, because organic carbon hosted in hydrophobic residual coal matter may have limited short-term transfer into the aqueous phase. Future source-term release assessment and leaching risk identification of CG should evaluate COD together with leachate DOC, solid-phase organic carbon occurrence, and possible inorganic reducing substances. This would help more accurately identify the sources and release potential of mobile organic components or reducing substances.

In addition, all leaching experiments in this study were conducted at room temperature. Under field conditions, seasonal temperature variations and the structure of CG piles may generate spatial and temporal temperature differences within the piles. Microbial processes were also not incorporated into either the experimental systems or the PHREEQC model. Temperature and microbial activity may affect mineral dissolution, organic matter degradation, sulfur transformation, and interfacial adsorption–desorption processes. Therefore, the present results may not fully represent the release and transformation of COD-related components, SO_4_^2−^, and F^−^ under field conditions and over longer timescales. Future studies will combine field monitoring with pile-scale experiments to further evaluate the effects of temperature variation and microbial processes on the long-term release and transformation of non-metallic constituents from CG.

## 4. Conclusions

This study demonstrated that the aqueous mobilization of oxidizable components contributing to COD, together with the release of SO_4_^2−^ and F^−^ from CG, was dependent on sample source and leaching stage. The batch tests identified differences among the 20 samples in COD response and the release potential of SO_4_^2−^ and F^−^, while the column tests revealed distinct L/S-dependent patterns. COD levels and SO_4_^2−^ concentrations were highest during the initial leaching stage and subsequently decreased, whereas F^−^ exhibited delayed and persistent release.

In the batch tests, COD levels ranged from 4.6 to 68.0 mg/L, while the initial column-leachate levels of CG-A and CG-B reached 91.4 and 186.6 mg/L, respectively, at L/S = 0.2 L/kg. Solid-phase TOC was not directly related to aqueous organic-carbon release: samples containing 391–703 mg/g TOC produced only 2.6–6.1 mg/L DOC, whereas a sample containing 3.8 mg/g TOC generated 78.9 mg/L DOC and 106.3 mg/L COD. ToF-SIMS detected aliphatic organic fragments, including C_2_H_5_^−^, C_3_H_5_^−^, and C_3_H_7_^−^, associated with Al-, Si-, and K-bearing surfaces, and some of these signals weakened or were redistributed after leaching. Collectively, these results indicate that the COD response was more closely associated with the aqueous accessibility of surface-associated organic components than with the bulk TOC content of CG and may also have been affected by inorganic reducing species.

For SO_4_^2−^, batch-leachate concentrations were generally 55–170 mg/L, with a maximum of approximately 317 mg/L, whereas the initial column-leachate concentrations reached 830.5, 3074, and 1872 mg/L for CG-A, CG-B, and CG-C, respectively, at L/S = 0.2 L/kg. These concentrations decreased rapidly as L/S increased, indicating pronounced early-stage flushing of readily releasable sulfate-related components. Within the PHREEQC-XGBoost-SHAP framework, the sulfate source-term coefficient accounted for 69.4–76.9% of the modeled influence at L/S = 0.5–2.0 L/kg; at L/S = 10.0 L/kg, its relative influence decreased to 49.7%, while that of HFO surface complexation increased to 49.8%. The modeled control on SO_4_^2−^ release therefore shifted from the initial sulfate source term toward an approximately equal influence of the source term and surface complexation during the later leaching stage, supporting intensive monitoring of SO_4_^2−^ during early rainfall infiltration and continued attention during long-term leaching.

For F^−^, batch-leachate concentrations ranged from 0.14 to 1.29 mg/L, while column-leachate concentrations remained at approximately 1.6–2.3 mg/L in CG-A, 0.3–1.8 mg/L in CG-B, and 0.40–0.83 mg/L in CG-C. Unlike COD and SO_4_^2−^, F^−^ was not dominated by initial flushing but persisted throughout the column experiment; correspondingly, ToF-SIMS showed no marked decrease in the F^−^ signal after leaching and revealed its partial spatial association with Ca. The F^−^ source-term coefficient accounted for 97.0–97.9% of the modeled influence over L/S = 0.5–10.0 L/kg. These experimental and modeling results are consistent with the continuing availability of releasable fluorine-bearing components during leaching and highlight the need for long-term F^−^ monitoring at CG disposal and backfill sites.

## Data Availability

The raw data supporting the conclusions of this article will be made available by the authors on request.

## Supplementary Materials

The following supporting information can be downloaded at: https://www.mdpi.com/article/10.3390/toxics14070635/s1, Text S1: Parameter definitions, source-term equations, and ranges; Text S2: PHREEQC settings; Text S3: XGBoost surrogate model settings; Text S4: SHAP contribution analysis and spearman validation; Figure S1: XRD patterns of coal gangue samples; Figure S2: Dynamic SHAP Feature Importance Across Leaching Stages; Table S1: Predictive performance of the XGBoost surrogate model for SO_4_^2−^ and F^−^ concentrations; Table S2: L/S-dependent pH profiles of leachates from the Method 1314 column tests.

## Abbreviations

The following abbreviations are used in this manuscript:

CG	coal gangue
COD	chemical oxygen demand
DOC	dissolved organic carbon
TOC	total organic carbon
ToF-SIMS	time-of-flight secondary ion mass spectrometry
PHREEQC	geochemical modeling software
XGBoost	extreme gradient boosting
SHAP	SHapley Additive exPlanations
LHS	Latin hypercube sampling
L/S	liquid-to-solid ratio
HFO	hydrous ferric oxide

## References

[1] International Energy Agency Statistics (2024). Coal 2024: Analysis and Forecast to 2027.

[2] Li D., Wu D., Xu F., Lai J., Shao L. (2018). Literature Overview of Chinese Research in the Field of Better Coal Utilization. J. Clean. Prod..

[3] Dai S., Sun F., Wang L., Wang L., Zhang R., Guo H., Xing Y., Gui X. (2023). A New Method for Pre-Enrichment of Gallium and Lithium Based on Mode of Occurrence in Coal Gangue from Antaibao Surface Mine, Shanxi Province, China. J. Clean. Prod..

[4] Cheng Y., Ma H., Chen H., Wang J., Shi J., Li Z., Yu M. (2018). Preparation and Characterization of Coal Gangue Geopolymers. Constr. Build. Mater..

[5] Li J., Wang J. (2019). Comprehensive Utilization and Environmental Risks of Coal Gangue: A Review. J. Clean. Prod..

[6] Stracher G.B., Taylor T.P. (2004). Coal Fires Burning out of Control around the World: Thermodynamic Recipe for Environmental Catastrophe. Int. J. Coal Geol..

[7] Zhang J., Zhang Y., He G., Wan X., Chen X., Zhao J. (2024). Wind Speed Effect on Infrared-Image-Based Coal and Gangue Recognition with Liquid Intervention in LTCC. J. Clean. Prod..

[8] Li J., Li X., Huang Y., Zhang D., Lv F., Huang P. (2024). Dynamic Leaching Behaviors of Heavy Metals from Recycled Coal Gangue Aggregate under Loading Conditions during Solid Backfill Mining. Environ. Pollut..

[9] Dong J., Li J., Huang Y., Zhong J., Dun K., Wu M., Zhang L., Chen Q., Pan B. (2024). Understanding the Release, Migration, and Risk of Heavy Metals in Coal Gangue: An Approach by Combining Experimental and Computational Investigations. J. Hazard. Mater..

[10] Xu S., Yang Y., Li W., Lu Y., Yang H., Huang Q., Die Q. (2026). Leaching Mechanisms of Potentially Toxic Elements from Coal Gangue: Thermodynamic, Kinetic, and Colloidal Effects—ScienceDirect. Environ. Pollut..

[11] Cheng L., Jiang C., Li C., Zheng L. (2022). Tracing Sulfate Source and Transformation in the Groundwater of the Linhuan Coal Mining Area, Huaibei Coalfield, China. Int. J. Environ. Res. Public Health.

[12] Dong Y., Gao Z., Di J., Wang D., Yang Z., Guo X., Li Y., Zhu X., Wang G. (2023). Remediation of Acid Mine Drainage in the Haizhou Open-Pit Mine through Coal-Gangue-Loaded SRB Experiments. Sustainability.

[13] Yuan Z., Shi S., Wu X., Wang S., Tian W. (2024). Aliphatic and Polycyclic Aromatic Compounds in Coal and Coal-Based Solid Wastes: Relationship with Coal-Forming Paleoenvironment and Implications for Environmental Pollution. Sci. Total Environ..

[14] Burgos C., Chamorro S., Monsalves N., Gómez G., Vidal G. (2025). Assessment of Acute Toxicity of Acid Mine Drainage via Toxicity Identification Evaluation (TIE) Using *Daphnia magna* and *Chlorella vulgaris*. Water.

[15] Bhuiyan M.A.H., Islam M.A., Dampare S.B., Parvez L., Suzuki S. (2010). Evaluation of Hazardous Metal Pollution in Irrigation and Drinking Water Systems in the Vicinity of a Coal Mine Area of Northwestern Bangladesh. J. Hazard. Mater..

[16] Duan C., Zhou C., Dong L., Zhao Y., Liu Q. (2018). A Novel Dry Beneficiation Technology for Pyrite Recovery from High Sulfur Gangue. J. Clean. Prod..

[17] Chen G., Ye Y., Yao N., Hu N., Zhang J., Huang Y. (2021). A Critical Review of Prevention, Treatment, Reuse, and Resource Recovery from Acid Mine Drainage. J. Clean. Prod..

[18] Naidu G., Ryu S., Thiruvenkatachari R., Choi Y., Jeong S., Vigneswaran S. (2019). A Critical Review on Remediation, Reuse, and Resource Recovery from Acid Mine Drainage. Environ. Pollut..

[19] Wu Z., Gao X., Li C., Huang H., Bai X., Zheng L., Shi W., Han J., Tan T., Chen S. (2025). Mechanisms and Genesis of Acidic Goaf Water in Abandoned Coal Mines: Insights from Mine Water–Surrounding Rock Interaction. Minerals.

[20] Gao X., Hu Y., Li C., Dai C., Li L., Ou X., Wang Y. (2016). Evaluation of Fluorine Release from Air Deposited Coal Spoil Piles: A Case Study at Yangquan City, Northern China. Sci. Total Environ..

[21] Yang N., Tang S., Zhang S., Huang W., Chen P., Chen Y., Xi Z., Yuan Y., Wang K. (2017). Fluorine in Chinese Coal: A Review of Distribution, Abundance, Modes of Occurrence, Genetic Factors and Environmental Effects. Minerals.

[22] Yadav K., Raphi M., Jagadevan S. (2021). Geochemical Appraisal of Fluoride Contaminated Groundwater in the Vicinity of a Coal Mining Region: Spatial Variability and Health Risk Assessment. Geochemistry.

[23] Liu X., Hu Z., Gao J. (2025). Assessment of Soil and Groundwater Pollution Risks from Coal Gangue Backfilling: A Case Study in Inner Mongolia Mining Areas. Environ. Res..

[24] da Silva E.B., Li S., de Oliveira L.M., Gress J., Dong X., Wilkie A.C., Townsend T., Ma L.Q. (2018). Metal Leachability from Coal Combustion Residuals under Different pHs and Liquid/Solid Ratios. J. Hazard. Mater..

[25] Zhao L., Zhang Y., Du C., Jiang B., Wei L., Li Y. (2021). Characterization of Dissolved Organic Matter Derived from Coal Gangue Packed in Underground Reservoirs of Coal Mines Using Fluorescence and Absorbance Spectroscopy. Environ. Sci. Pollut. Res..

[26] Underwood T., Erastova V., Greenwell H.C. (2016). Wetting Effects and Molecular Adsorption at Hydrated Kaolinite Clay Mineral Surfaces. J. Phys. Chem. C.

[27] Song X., Sun Y., Wang H., Huang X., Han Z., Shu Y., Wu J., Zhang Z., Zhong Q., Li R. (2025). Uncovering Soil Heavy Metal Pollution Hotspots and Influencing Mechanisms through Machine Learning and Spatial Analysis. Environ. Pollut..

[28] Alam S.M.K., Li P., Rahman M., Fida M., Elumalai V. (2025). Key Factors Affecting Groundwater Nitrate Levels in the Yinchuan Region, Northwest China: Research Using the eXtreme Gradient Boosting (XGBoost) Model with the SHapley Additive exPlanations (SHAP) Method. Environ. Pollut..

[29] (2007). Solid Waste—Extraction Procedure for Leaching Toxicity-Sulphuric Acid & Nitric Acid Method.

[30] (2017). Water Quality—Determination of the Chemical Oxygen Demand-Dichromate Method.

[31] (2009). Water Quality—Determination of Fluoride-Fluoride Reagent Spectrophotometric Method.

[32] (2007). Water Quality—Determination of Sulfate-Barium Chromate Spectrophotometric Method.

[33] Ai M., Cheng W., Wang Z., Huang X. (2025). Recovery of Lithium from Coal Gangue, with an Emphasis on Lithium Occurrence, Roasting Pretreatment and Leaching. Miner. Eng..

[34] (2002). Environmental Quality Standards for Surface Water.

[35] Chen Y., Glaus M.A., Loon L.R.V., Mader U. (2018). Transport of Low Molecular Weight Organic Compounds in Compacted Illite and Kaolinite. Chemosphere.

[36] Evangelou V.P., Zhang Y.L. (1995). A Review: Pyrite Oxidation Mechanisms and Acid Mine Drainage Prevention. Crit. Rev. Environ. Sci. Technol..

[37] Sun Y., Zhang M., Luo D., Long Q., Guo W., Hou J., Chang L., Han Y., Peng X., Tao Y. (2026). The pH-Driven Distribution and Migration of Phosphate, Fluoride and Metals/Metalloids in Phosphogypsum Stacks: Insights from Southwest China. Molecules.

[38] Scanlon B.R., Stonestrom D.A., Reedy R.C., Leaney F.W., Gates J., Cresswell R.G. (2009). Inventories and Mobilization of Unsaturated Zone Sulfate, Fluoride, and Chloride Related to Land Use Change in Semiarid Regions, Southwestern United States and Australia. Water Resour. Res..

