# Supplementary material for: Source-Term Release Behavior and Mechanisms of Non-Metallic Leaching Parameters from Coal Gangue: COD, Sulfate, and Fluoride
Source: Toxics. 2026 Jul 21;14(7):635. doi: 10.3390/toxics14070635 (PMC13418881; doi:10.3390/toxics14070635)
Supplement: Supplementary file 1 [file toxics-14-00635-s001.zip › toxics-4398061-supplementary.pdf]

Supplementary material

S1 Parameter definitions, source-term equations, and ranges

Three parameters, $k_{SO_4^{2-}}$, k_{F^-}, and $p_{m,HFO}$, were selected as the main model inputs. The source-term concentrations of SO_4^{2-} and F^- entering the PHREEQC model were described using a first-order exponential function, with cumulative liquid-to-solid ratio (L/S) as the leaching-progress variable:

$$C_i(x) = \left(C_{0,i} - C_{eq,i}\right)e^{-k_i(x-x_0)} + C_{eq,i} \quad \text{(S1)}$$

where $C_i(x)$ is the source-term concentration of component i at cumulative L/S x (mg/L); x is the cumulative L/S (L/kg); x_0 is the initial anchor point of 0.2 L/kg; $C_{0,i}$ is the initial concentration at x_0; $C_{eq,i}$ is the prescribed long-term equilibrium or background concentration; and k_i is an effective first-order coefficient defined with respect to cumulative L/S. Because the exponential term must be dimensionless, the unit of k_i is kg/L.

The parameter $k_{SO_4^{2-}}$ describes the rate at which the source-term concentration of readily releasable sulfate-related components decreases toward its long-term value as cumulative L/S increases. The parameter k_{F^-} describes the rate at which the F^- source-term concentration approaches its prescribed long-term value. A larger k_i value indicates a more rapid approach to the corresponding long-term concentration with increasing L/S. These parameters are effective empirical coefficients defined on the L/S scale. They should not be interpreted as time-based mineral dissolution rate constants or thermodynamic equilibrium constants.

The parameter $p_{m,HFO}$ represents the logarithm of the total amount of reactive hydrous ferric oxide (HFO) surface sites:

$$n_{HFO} = 10^{p_{m,HFO}} \quad \text{(S2)}$$

where n_{HFO} is the total HFO surface-site amount expressed as mol sites/kg dry solid. The total HFO surface sites were divided into weak and strong sites at a ratio of 98:2:

$$n_w = 0.98 n_{HFO} \quad \text{(S3)}$$

$$n_s = 0.02 n_{HFO} \quad \text{(S4)}$$

where n_w and n_s are the amounts of weak and strong HFO complexation sites,

respectively. Thus, $p_{m,HFO}$ characterizes the surface complexation capacity of reactive metal oxides and represents the potential influence of adsorption–desorption processes on aqueous SO_4^{2-} and F^- concentrations.

Latin hypercube sampling was conducted over ranges of 0.1–5.0 kg/L for both $k_{SO_4^{2-}}$, k_{F^-}, and −4.0 to −1.0 for $p_{m,HFO}$. The latter range corresponds to approximately 10^{-4}–10^{-1} mol sites/kg dry solid. The L/S = 0.2 L/kg point was used as the initial concentration anchor. Because the predicted concentration at this point was fixed by $C_{0,i}$ and was independent of k_i, it was excluded from SHAP and Spearman analyses to prevent the initial constraint from being interpreted as parameter influence.

S2 PHREEQC settings

The PHREEQC model was used to simulate carbonate buffering and surface complexation redistribution of SO_4^{2-} and F^- during coal gangue–water interaction. The minteq.v4.dat database was used. The simulation temperature was set to 25 °C, and the initial pH was set to 6.0. Calcite was defined as the main carbonate buffering phase using the *EQUILIBRIUM PHASES* module. This setting was used to represent the regulation of solution pH and ionic speciation by carbonate minerals during leaching. HFO surface sites were defined using the SURFACE module to simulate the adsorption–desorption of SO_4^{2-} and F^- on reactive metal oxide surfaces. After each PHREEQC calculation, the total amounts of F and S(6) in solution were extracted as model outputs. S(6) was then converted to SO_4^{2-} concentration.

S3 XGBoost surrogate model settings

Latin hypercube sampling (LHS) was used to generate 10,000 parameter combinations of $k_{SO_4^{2-}}$, k_{F^-}, and $p_{m,HFO}$. PHREEQC was then run in batch mode to obtain simulated SO_4^{2-} and F^- concentrations at different L/S stages. The simulation points were consistent with the Method 1314 column leaching experiment, including 0.2, 0.5, 1.0, 1.5, 2.0, 4.5, 5.0, 9.5, and 10.0 L/kg.

The XGBoost surrogate model used $k_{SO_4^{2-}}$, k_{F^-}, and $p_{m,HFO}$ as input variables and PHREEQC-simulated SO_4^{2-} and F^- concentrations as output variables. Before model training, the output concentrations were log10-transformed and standardized to reduce

the influence of scale differences between SO_4^{2-} and F^- concentrations. The dataset was divided into training, validation, and test sets at proportions of 70%, 15%, and 15%, respectively.

A multi-output regression strategy was used for XGBoost. The main hyperparameters were set as follows: n estimators = 200, learning rate = 0.1, max depth = 6, subsample = 0.8, and colsample bytree = 0.8. Model performance was evaluated using R^2, RMSE, and MAE. R^2 was mainly calculated on the log-transformed concentration scale, whereas RMSE and MAE were also reported on the original concentration scale to reflect the actual magnitude of prediction errors. The model validation results are provided in **Table S1.**

S4 SHAP contribution analysis and spearman validation

The trained XGBoost surrogate model was interpreted using SHAP to calculate the relative contributions of $k_{SO_4^{2-}}$, k_{F^-}, and $p_{m,HFO}$ to the predicted SO_4^{2-} and F^- concentrations. For each pollutant and each L/S point, the mean absolute SHAP value of each input parameter was first calculated and then normalized as the relative contribution:

$$RC_j = \frac{mean(|SHAP_j|)}{\sum mean(|SHAP_j|)} \times 100\% \quad \text{(S5)}$$

where RCj is the relative contribution of parameter j, and mean($|SHAPj|$) is the mean absolute SHAP value of parameter j for a given pollutant and L/S point. $k_{SO_4^{2-}}$ represents the release/elution control of SO_4^{2-}-related components, k_{F^-} represents the release control of fluorine-bearing components, and c represents HFO surface complexation capacity and adsorption–desorption processes. To avoid interference from the initial concentration anchor, SHAP analysis was performed only for L/S = 0.5–10.0 L/kg.

To examine the statistical consistency of the SHAP interpretation, Spearman rank correlation analysis was used to calculate the relationships between input parameters and PHREEQC-simulated concentrations. The L/S = 0.2 L/kg point was also excluded from the Spearman analysis, which was performed on the log10-transformed

concentration scale. The SHAP contribution results, together with the direction of the Spearman correlations, were used to support the interpretation of the relative influence of the processes represented by different parameters on SO_4^{2-} and F^- release. It should be noted that the SHAP relative contributions represent the relative influence of input parameters on XGBoost surrogate model predictions. They do not represent absolute mass-flux contributions of the corresponding mechanisms in the actual reaction processes.

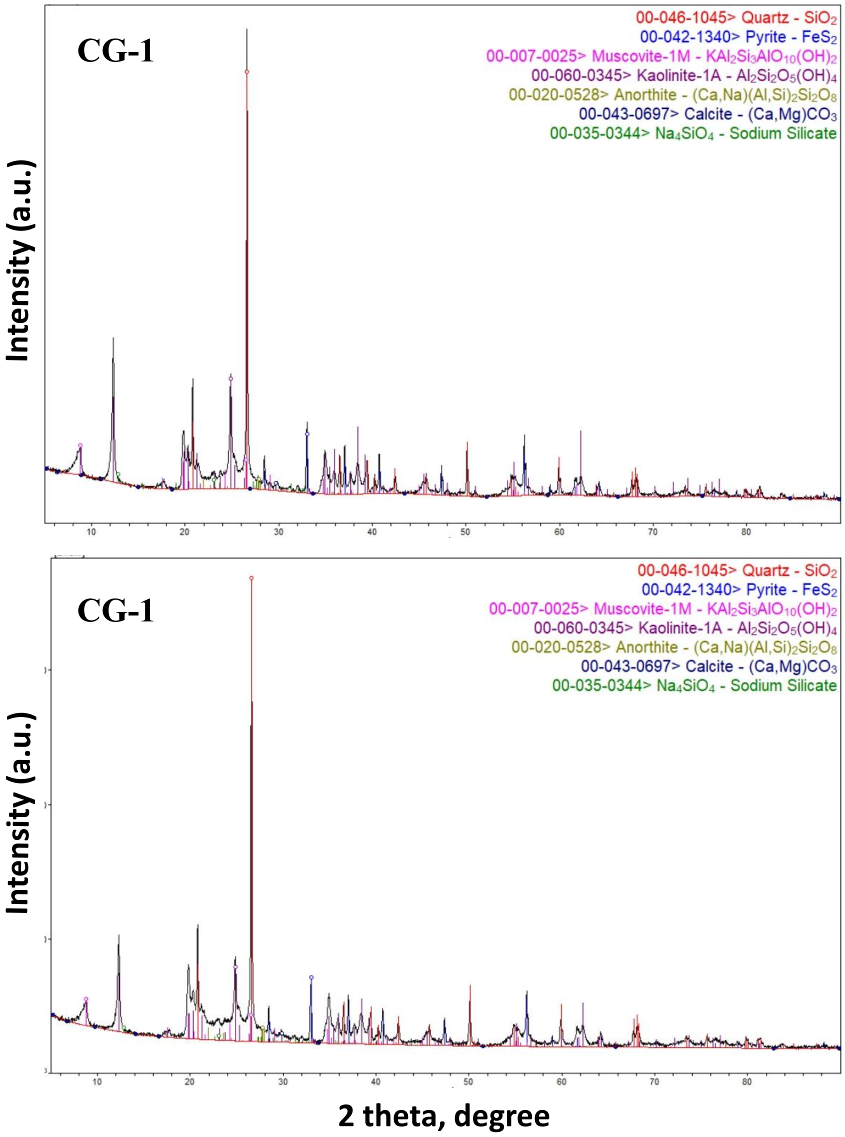

Figure S1 XRD patterns of coal gangue samples.

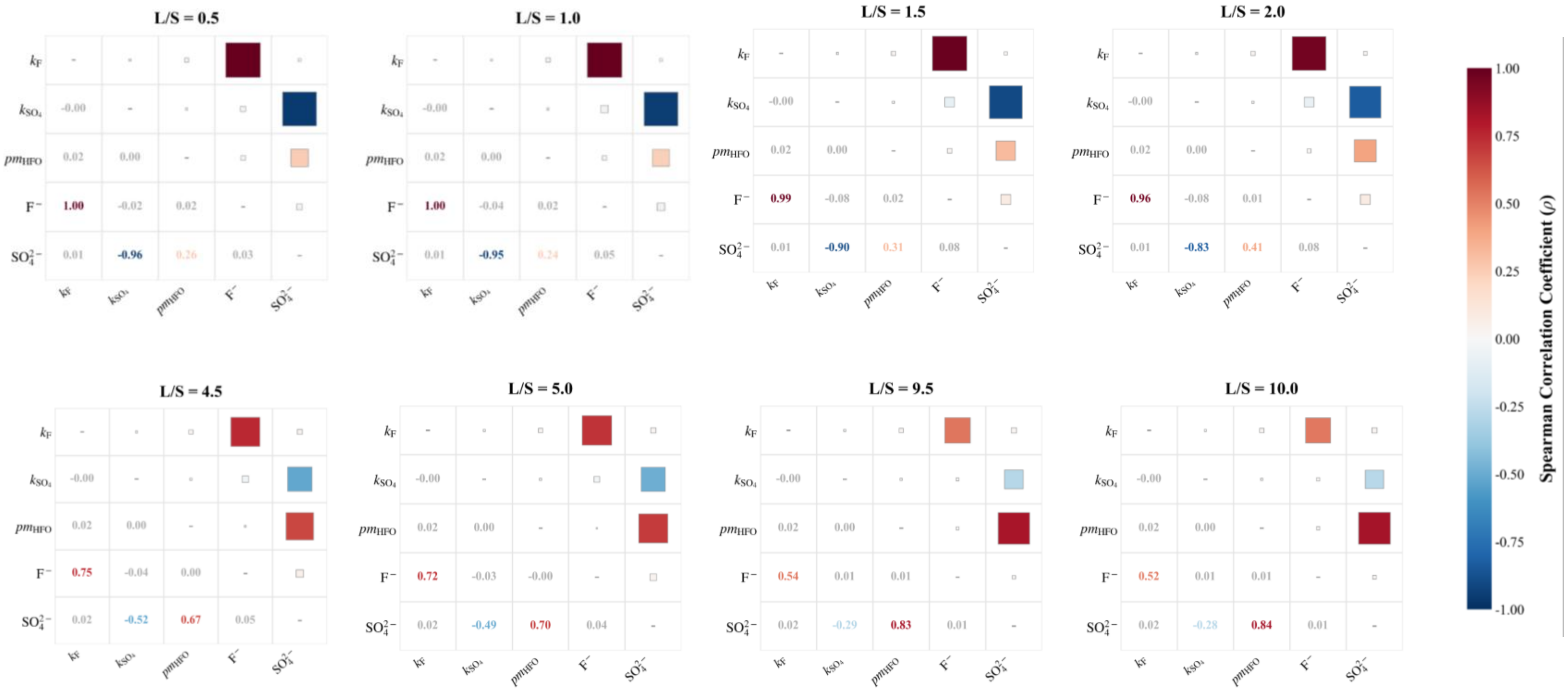

Figure S2 Dynamic SHAP Feature Importance Across Leaching Stages.

Table S1 Predictive performance of the XGBoost surrogate model for SO_4^{2-} and F^- concentrations.

Output variable	Dataset	R^2_{log10}	$RMSE_{log10}$	MAE_{log10}
	Training set	0.998	0.006	0.004
SO_4^{2-}	Validation set	0.991	0.013	0.007
	Test set	0.995	0.013	0.007
	Training set	0.981	0.018	0.008
F^-	Validation set	0.975	0.021	0.004
	Test set	0.977	0.022	0.004

Table S2. L/S-dependent pH profiles of leachates from the Method 1314 column tests.

L/S	CGA-pH	CGB-pH	CGC-pH
0.2	8.36	7.36	8.03
0.5	8.6	7.28	7.99
1	8.62	7.32	8
1.5	8.54	7.3	7.95
2	8.03	7.27	7.84
4.5	8.19	7.26	7.86
5	8.07	7.23	7.81
9.5	8.04	7.21	7.8
10	8.06	7.19	7.77
